# STAT3 and p53: Dual Target for Cancer Therapy

**DOI:** 10.3390/biomedicines8120637

**Published:** 2020-12-21

**Authors:** Thu-Huyen Pham, Hyo-Min Park, Jinju Kim, Jin Tae Hong, Do-Young Yoon

**Affiliations:** 1 Department of Bioscience and Biotechnology, Konkuk University, Seoul 05029, Korea; huyenpham@konkuk.ac.kr (T.-H.P.); wkd910222@konkuk.ac.kr (H.-M.P.); jinjukim78@konkuk.ac.kr (J.K.); 2 College of Pharmacy and Medical Research Center, Chungbuk National University, Chungbuk 28160, Korea; jinthong@chungbuk.ac.kr

**Keywords:** STAT3, wild-type p53 (wtp53), mutant p53 (mtp53), feedback regulation, drug resistance, cancer therapy

## Abstract

The tumor suppressor p53 is considered the “guardian of the genome” that can protect cells against cancer by inducing cell cycle arrest followed by cell death. However, STAT3 is constitutively activated in several human cancers and plays crucial roles in promoting cancer cell proliferation and survival. Hence, STAT3 and p53 have opposing roles in cellular pathway regulation, as activation of STAT3 upregulates the survival pathway, whereas p53 triggers the apoptotic pathway. Constitutive activation of STAT3 and gain or loss of p53 function due to mutations are the most frequent events in numerous cancer types. Several studies have reported the association of STAT3 and/or p53 mutations with drug resistance in cancer treatment. This review discusses the relationship between STAT3 and p53 status in cancer, the molecular mechanism underlying the negative regulation of p53 by STAT3, and vice versa. Moreover, it underlines prospective therapies targeting both STAT3 and p53 to enhance chemotherapeutic outcomes.

## 1. Introduction

Cancer is one of the leading causes of death worldwide, which was responsible for approximately 9.6 million cancer deaths in 2018 [1]. Targeted chemotherapy is a common method of cancer treatment in which the molecular pathways related to cancer growth or metastasis are blocked using targeted drugs. Molecularly targeted drugs are less toxic and more effective than conventional drugs because they are administered at lower doses than the higher tolerated dose of the latter [2]. However, both types of drugs suffer from problems associated with cellular resistance, which reduces their efficacy [3]. In addition, chemoresistance is often associated with transformation of tumors into more aggressive and/or metastatic forms. 

Signal transduction and activator of transcription (STAT) 3 is a member of the STAT family, comprising seven transcription factors (STAT 1, 2, 3, 4, 5a, 5b, 6) [4]. It was discovered by two independent groups [5,6] and has been of particular interest due to its role in the regulation of cellular signaling, especially in cancers. STAT3 is constitutively active in several cancers such as breast, lung, ovarian, colorectal, cervical, gastric, and prostate cancers, and head and neck squamous cell carcinoma [7,8,9,10,11,12,13]. Despite the multifaceted function of STAT3 in cancer, growing evidence has revealed that constitutive activation of STAT3 contributes to cancer cell proliferation and that aberrant STAT3 activation is associated with tumor malignancy [14,15,16]. 

*TP53* (tumor protein p53) is one of the most well-studied tumor suppressor genes. Owing to its pivotal role in protection against malignancies, wild-type p53 (wtp53) has long been called the “guardian of the genome” [17]. It is well known that p53 suppresses tumor formation and renders protection against DNA damage by inducing cell cycle arrest, DNA repair, or apoptosis [18]. Mutation of p53 is often observed in cancer, especially in late events in malignant progression [19,20]. 

Tumor cell proliferation and survival involve downregulation of wtp53 expression as well as increase in STAT3 activity. In contrast, wtp53 reduces STAT3 phosphorylation and DNA-binding activity in breast and prostate cancer cells [21,22]. In addition, another report revealed that STAT3 activity suppresses *TP53* expression [23]. Therefore, wtp53 and activated STAT3 negatively regulate each other. This adverse regulation can be explained by the opposing biological roles of both factors, as activated STAT3 functions as an oncogene [24], whereas wtp53 functions as a tumor suppressor [25]. Consequently, normal cells might have evolved mechanisms to adjust STAT3 and p53 expression for necessary cell proliferation conditions, whereas tumor cells might exploit such negative regulation for survival [23]. During the early stage of progression, tumors grow preferentially via STAT3-regulated signaling [26]. Although mutations of p53 have been reported to occur early and involve in tumor initiation, it appears that p53 mutations in certain cancers could be developed late and contribute significant roles in advanced stages of tumorigenesis [27]. Furthermore, the loss of wtp53 function along with the accumulation of mutated p53 (mtp53) can support STAT3-mediated tumor cell survival and expansion [28,29,30]. 

Several inhibitors targeting either STAT3 or p53 are under clinical trials, but their success has been limited because of resistance to targeted cancer therapy [31,32]. Resistance often occurs due to the complexity of cancer signaling pathways, making it difficult for single-target inhibitors to achieve satisfactory clinical outcomes; hence, a combinational therapy co-targeting STAT3 and p53 could overcome drug resistance. The present review provides our current understanding of two well-known targets for cancer therapy, STAT3 and p53, regarding the interaction between them as well as the potential underlying mechanisms. In addition, we have summarized the status of STAT3 and p53 in different cancer cell types and highlighted the potential therapies that target both factors to improve the efficacy of cancer prevention.

## 2. Role of STAT3 Signaling in Cancer

### 2.1. Activation and Regulation of STAT3

STAT3 is maintained as an inactive homodimer in the cytoplasm of nonstimulated cells. It forms a stable dimer to translocate into the nucleus of stimulated cells and acts as a transcription factor for numerous targeted genes. Activation of STAT3 is induced by various cytokines (interleukin (IL)-6, type I interferons) and growth factors (epidermal growth factor (EGF), platelet-derived growth factor (PDGF)) through receptors (EGFR, PDGFR) and Janus kinase (JAK) signaling pathway [33,34,35,36,37,38], or through oncogenic proteins (Ras, protein kinase C (PKC)) [39,40,41]. It is stringently controlled by several negative regulators, including phosphatases (Src homology region 2 (SHP2), phosphatase and tensin homolog (PTEN), CD45) [42,43,44], suppressor of cytokine signaling proteins (SOCS), mainly SOCS3 [45], and protein inhibitors of activated STAT (PIAS) proteins, particularly PIAS3 [46]. 

STAT3 can be activated by two major mechanisms: nuclear activation upon tyrosine phosphorylation (Tyr705) and mitochondrial activation (mitoSTAT3) upon serine phosphorylation (Ser727) [47]. The phosphorylation of STAT3 at Tyr705 is primarily regulated by JAK2, IL-6, and EGF, whereas phosphorylation at Ser727 is commonly regulated by PKC, mitogen-activated protein kinases (MAPKs), and cyclin-dependent kinase (CDK) [39,48]. Phosphorylation of STAT3 at Tyr705 site has been studied extensively as it leads to nuclear translocation, DNA binding activities, and transcription of target genes [48]. It has been shown that mitoSTAT3 along with phosphorylated Ser727 can promote tumor growth and metastasis [47]. Phosphorylation of STAT3 at Ser727 supports or represses the transcriptional activity of STAT3 in the presence of phosphorylated Tyr705 [49]. The actual effects of Ser727 phosphorylation remain somewhat controversial. 

### 2.2. Function as an Oncogene

STAT3, like other STAT proteins, was initially characterized for its role in cytokine signaling and was then classified as an oncogene for the following reasons. First, it is constitutively active in several tumor samples and is correlated with high metastatic threat and poor survival consequences [8,11,13,38,50]. Aberrant persistent STAT3 activity has been observed in various hematological and solid cancers [24]. Noticeably, constitutive STAT3 activity is frequently found in triple negative breast cancers, and in more than 40% of all breast cancers [51]. In normal cells, STAT3 is activated for a temporary duration from a few minutes to several hours [49]. The oncogenic role of STAT3 in gliomas is consistent with the observation that STAT3 activation is rarely detected in normal brain tissue [52]. Second, STAT3 acts as a transcription factor that activates several downstream target genes that are involved in multiple steps of metastasis, including invasion, cell survival, self-renewal, angiogenesis, and tumor-cell immune evasion [53]. It also localizes in the mitochondria and supports gene regulation [47]. Third, STAT3 is directly associated with oncogenic signaling and responses to specific oncogenic kinases, such as SRC, ABL, FPS, and JAK2 [54,55]. STAT3 can activate transcription in the absence of tyrosine phosphorylation by interacting with nuclear factor-κB (NF-κB) subunits to induce specific cancer genes [54]. STAT3 has been reported as a part of the JAK2/STAT3/STAT5/PD-L1 axis which can drive immune escape in myeloproliferative neoplasms [55]. Fourth, blocking STAT3 activity decreases cellular transformation in SRC-transformed cell lines [56]. Mutated STAT3 construct (STAT3C), which constitutively forms dimers in normal mouse fibroblasts, forms tumors when transplanted into nude mice. This STAT3C construct was found to drive tumor formation in a variety of cell types by upregulating important oncogenic and angiogenic factors such as matrix metalloproteinase MMP-9, vascular endothelial growth factor (VEGF). Although some evidence raised the question about the multifaceted function of STAT3 as it exerted a normal role in immunosuppressive cells [57], growth inhibitory effect in prostate cancer cells [58], and tumor suppressing functions in some cases [59], most observations demonstrated the major role of constitutively active STAT3 in tumorigenesis.

### 2.3. Targeting STAT3 for Cancer Therapies

Several strategies have been established to inhibit STAT3 signaling, including: (i) downregulating the upstream regulators, (ii) targeting the STAT3 SH2 domain, (iii) blocking the STAT3 DNA-binding domain, (iv) inhibiting the STAT3 N-terminal domain, (v) suppressing the STAT3 mRNA, and (vi) targeting the STAT3 endogenous negative regulators [60]. Direct inhibitors target the SH2 domain (Stattic, S3I-201 and derivatives, OPB-31121, OPB-51602), the DNA-binding domain (Decoy oligonucleotides [ODNs]), the N-terminal domain (ST3-HA2A), or the STAT3 mRNA (AZD9150) to regulate STAT3 activation [61,62,63,64,65,66,67]. Indirect inhibitors target the upstream regulators of the STAT3 signaling pathway (IL-6, RTK, JAK, SRC, BCR-ABL), such as siltuximab, sunitinib, sorafenib, ruxolitinib, bosutinib, or the endogenous STAT3-negative regulators (AdCN305-cppSOCS3 targeting SOCS3) [68,69,70,71,72,73]. The current promising direct STAT3 inhibitors which have entered clinical trials include STAT3 antisense-based AZD9150 (Phase I in hepatocellular carcinoma metastatic, Phase II in advanced cancers), OPB-31121 (Phase I in advanced cancers, phase I/II in hepatocellular carcinoma), OPB-51602 (Phase I in advanced cancers, hematologic malignancies), OPB-111077 (Phase I in solid tumors, leukemia), STAT3 decoy (Early phase I in head and neck cancer).

Feedback activation of STAT3 plays an important role in mediating drug resistance to various conventional chemotherapies and molecularly targeted therapies [32]. The long term activation of tyrosine kinases in malignant tumors can lead to constitutive activation of STAT3, which may not only provide advantages of growth and accumulation of tumor cells, but also confers resistance to conventional therapies that rely on apoptotic machinery to get rid of tumor cells [21]. The downstream outcomes of STAT3 activation supporting tumorigenesis consist of deregulation of cell cycle progression and protection against apoptosis [21]. For example, persistent activation of STAT3 can resist apoptosis in human myeloma cells [74], fibroblasts [75], breast cancer [76], and gastric cancer [13]. 

As stated above, once activated by phosphorylation at Tyr705, STAT3 forms a dimer and translocates into the nucleus. Hence, drugs targeting the dimeric form of STAT3 are expected to be useful for tumors that rely on STAT3 activation. The SH2 domain is necessary for STAT3 dimer formation and phosphorylation which are recruited to tyrosine-phosphorylated receptor complexes; thus, targeting the SH2 domain is a prospective approach. Some SH2 domain inhibitors have been used in preclinical research (S3I-201 and derivatives) or entered clinical trials (OPB-31121, OPB-51602) for hematologic cancer treatment [77]. However, STAT3 interacts with NF-κB subunits in the absence of Tyr705 phosphorylation or is modified at other sites such as Ser727 to activate transcription [49,78]. It has been reported that nuclear translocation and DNA binding of STAT3 can occur independently of their P-Y status [77]. These observations indicate that SH2 domain-targeting inhibitors may not be adequate to abolish STAT3 oncogenic functions totally, which may become the limitation of these compounds. Therefore, it is obvious that a drug targeting the dimer and its Tyr705 phosphorylation would probably be ineffective if a tumor does not depend solely on the dimeric STAT3 and Tyr705 site for modification.

In brief, several small molecules and inhibitors have been developed and have shown effects in cancer treatment in preclinical research; however, a small number of them could enter clinical trials due to the lack of efficacy issues.

## 3. The Contribution of p53 in Cancer

### 3.1. Role of wtp53

The p53 protein functions as a nuclear transcription factor in the form of a homotetramer and contributes to normal cellular processes. It is activated in response to stress conditions such as DNA damage, oncogenic stress, replicative stress, and hypoxia [25,79]. Activation of p53 is regulated through three basic steps: stabilization of p53, DNA binding to a specific sequence, and transcriptional initiation of target genes. Three major functions of p53 include growth arrest, DNA repair, and cell death (apoptosis and senescence). When there is DNA damage in the cell, the growth arrest stops the progression of the cell cycle, preventing replication of damaged DNA, and activating the transcription of proteins involved in DNA repair. If the DNA cannot be repaired, apoptosis or senescence would be the last step to avoid proliferation of cells containing abnormal DNA. Multiple p53-mediated downstream target genes have been implicated in apoptosis (*PUMA*, *NOXA*, *BAX*, *APAF1*, *FAS*), cell cycle arrest (*CDK1a*, *GADD45*, *14-3-3*), senescence (*PML*, *PAI-1*, *E2F7*), DNA damage repair (*POLK*, *MGMT*, *FANCC*, *ERCC5*, *XPC*, *DDB2*, *GADD45α*, *MSH2*, *POLH*), and DNA metabolism (*GLUT1/3/4*, *TIGAR*, *SLC7A11*) [25]. Metabolic dysfunction also triggers p53 expression, and it was reported that p53 could regulate metabolism by inducing ferroptosis, an iron-dependent regulated form of cell death, or autophagy cell death [25]. Furthermore, p53 is involved in other cellular processes, including cell differentiation and stem cell renewal [79]. p53 is essential for regulating DNA repair and cell division; hence, it has been described as the “guardian of the genome” [18].

### 3.2. Negative Regulation of wtp53

wtp53 is inactivated by negative regulators such as E3-ubiquitin ligases (mouse double minute 2 [MDM2], C-terminus of HSC70-interacting protein [CHIP], tripartite motif-containing 24 [TRIM24]), and asparaginase endopeptidase [31]. Under normal conditions, the protein level of p53 is low because of the feedback regulation between p53 and MDM2, an E3 ubiquitin-protein ligase [79]. MDM2 is the most recognized p53 inactivator. Cellular stress disrupts MDM2 binding to p53 by phosphorylation of both proteins and stimulates p53 acetylation, leading to p53 accumulation and activation [79]. p53 activates the MDM2 gene, and subsequently, the MDM2 protein directly binds to and triggers the degradation of p53 using the ubiquitin system. The constitutive expression of MDM2 is sufficient for maintaining a normal level of p53 protein. Thus, the feedback loop p53–MDM2 is critical for regulating p53 activity to protect cells against DNA damage induced by stress [31,80]. Another notable homolog of MDM2 is MDM4, which acts like MDM2 to inhibit p53 transcriptional activity. The different mechanism of MDM4 compared to MDM2 is due to the lack of intrinsic E3 ubiquitin activity; however, it can bind to MDM2 and trigger ubiquitylation of p53 [31]. 

### 3.3. p53 Mutations in Cancer—From Loss of Function to Gain of Function

Mutations in *TP53* are often present in nearly 50% of all human cancers [81]. Missense mutation, where a single amino acid is substituted within the DNA binding domain of *TP53*, especially at six hot-spot codons (R175, G245, R248, R249, R273, R282), is the most frequently found type of mutation (approximately 80–90%). Other mutations, including insertion, deletion, and nonsense, occur in a small number [31]. 

The common types of mutations affecting *p53* function are loss of function (LOF) and gain of function (GOF). The p53 LOF mutation was first proposed by Alfred G. Knudson in 1971 [82]. More than 90% of cancers with p53 mutations present loss of both functional alleles [31]. The most common cause of p53 LOF is a missense mutation in one allele that leads to the inactivation of *TP53*. Based on the loss of p53 functionality, damaged cells may transfer their mutations, without being repaired, to the next generation. The accumulation of deregulated p53 often leads to the formation of tumors.

GOF is described as the ability of mtp53 to be exerted in the absence of wtp53 co-expression [83]. This function includes the capacity to promote cell proliferation, invasion, and metastasis; inhibit apoptosis; and induce resistance to cancer treatments [31]. Notably, the GOF mutation is usually a hot-spot mutation and occurs at a higher frequency than expected [84]. Knock-in allele of some common p53 mutations within hot-spot codons, using a mouse model, demonstrated the GOF phenotype, which supported tumor development and metastasis [85]. A proposed mechanism by which mtp53 exerts GOF is the binding and modulation of the function of other transcriptional regulators such as p63, p73, NF-X, and NF-Y [83]. Another mechanism is the upregulation of chromatin regulatory enzymes such as MLL1, MLL2, and MOZ, which increase histone methylation and acetylation, subsequently promoting cancer cell growth [86]. 

Recently, p53 mutations were defined as separation of function (SOF) mutations [84]. SOF mutations produce stable proteins with loss of certain biochemical properties, but do not disrupt the other wild-type allele activities [84]. It has been shown that several *TP53* truncating mutations occur at the boundary of exon 6/exon 7, which induce cell proliferation and metastatic features in cancer cells. Particularly, these p53-exon-6 truncated proteins have molecular characteristics similar to those of the p53 alternative splice isoforms, and partially localize to the mitochondria to interact with cyclophilin D (CypD), a regulator of the mitochondrial permeability transition pore (MPTP) [84,87]. SOF mutations occur especially at hot-spot locations, and the total frequency is limited [84]. 

Mtp53 is more stable than wtp53 because it does not activate the expression of its negative regulator, MDM2, nor is it degraded by MDM2. In addition, mtp53 interacts with chaperones (heat shock protein (HSP)90, HSP70) to form a stable association that supports cancer cell survival under stress-induced conditions, and blockage of this mechanism elicits mtp53 degradation [88]. Therefore, in cancer cells, mtp53 may accumulate more extensively than wtp53 and exert its dominant negative effect against the wild-type function [89]. It has been shown that wtp53 and mtp53 are co-expressed at an equivalent level in vitro and in vivo [89]. Notably, the mtp53 allele is not generally carried in human nontransformed tissues and is found in patients with the rare disorder Li-Fraumeni Syndrome (LFS) [90]. Moreover, LFS patients would have one allele harboring wtp53 in untransformed tissues, whereas the majority of tumors upon transforming events maintain only the mutant allele [90]. This raises a question regarding the relationship between different *TP53* mutations and LFS patients. One explanation could be that during evolution or at an early stage of tumor generation, mtp53 is derived from one mutated allele co-existing with wtp53 from the other allele until the wild-type allele is totally lost by loss of heterozygosity (LOH), which results in the existence of only one mtp53 allele [90]. LFS patients hold different germline mutations in *TP53*; thus, they are susceptible to cancer development [84]. Consistent with this notion, LFS patients with the LOF *TP53* mutation would have tumors later in life, whereas the GOF *TP53* mutation group tends to acquire cancers in their inherited generation [91].

### 3.4. Mutant p53 and Cancer Therapy Resistance

Current strategies targeting p53 in cancer include two types: one targets wtp53 by blocking the degeneration of wtp53 or prolonging its cellular life and disrupting the interaction between wtp53 and its negative regulators MDM2/MDM4; the other targets mtp53 by destabilization of highly accumulated GOF p53 mutants and reactivation of mtp53 via recovery of the wild-type conformation and activity [31,92,93]. Other approaches that indirectly target mtp53 focus on the mtp53-specific downstream signaling pathways, the retaining G2 checkpoint on which a tumor depends, and the mtp53 interactors related to cancer progression [81]. 

Cancers harboring mtp53 are commonly characterized by serious metastasis and genomic instability; mtp53 is considered a “guardian of the cancer cell” [88]. A variety of p53 mutations produce different oncogenic activities to support tumor development. Generally, mtp53 core activities are recognized as the mirror basal function of the wtp53 counterpart and the adaptive ability to perform oncogenic function. p53 mutations have been linked to chemoresistance in breast, ovarian, lung, gastric, and colorectal cancers [94]. It is not only LOF but also GOF mutation forms that contribute to drug resistance. 

The mtp53 confers resistance to different MDM2 inhibitors, as these compounds mainly target wtp53 [95]. Another reason might be that MDM2 inhibitors cannot bind to MDM4, which is an MDM2 homolog with similarities in the N-terminal p53-binding domains; thus, most of the available MDM2 inhibitors lack activity against MDM4 [96]. For example, Nutlin-3a can activate wtp53 in cancer cells overexpressing MDM2 but not in cells overexpressing MDM4 [97]. Another problem with MDM2/MDM4 inhibitors is the unexpected increase in the expression levels of non-MDM2/MDM4 E3 ubiquitin ligases that may degrade wtp53 [98]. These MDM2 inhibitors would be effective mostly in wtp53 tumors because it is possible that p53 pathway restoration disrupts survival pathways and causes cancer cell death, although they also exert hematological toxicity as side effects during clinical trials [99,100]. Therefore, MDM2 antagonists might need to be better developed or used in combination with another method to increase specificity and reduce side effects.

Drug absorption and DNA repair changes are also possible mechanisms causing drug resistance in p53-based cancer therapy. For example, mtp53 stimulates the expression of ABCB1, an ABC transporter, and mediates drug efflux from cells in an ATP-dependent manner, conferring multidrug resistance [94]. Furthermore, p53 mutants disrupt critical DNA damage response pathways by interfering with binding of the MRE11–RAD50–NBS1 complex to the site of DNA damage, resulting in *ataxia telangiectasia mutated* (*ATM*) inactivation and genetic instability [101]. Notably, mtp53 recruits poly(ADP-ribose) polymerase 1 (PARP1), MCM4, and proliferating cell nuclear antigen (PCNA) to change chromatin structure and thus negatively regulates DNA repair while still allowing DNA replication to increase in breast cancer cells [102]. From these observations, it can be inferred that the indirect p53 inhibition approach could not satisfy drug treatment outcomes; hence, there is a need for a combination method that directly targets mtp53 as well as cancer-specific activation mechanisms.

## 4. STAT3 and p53 Feedback Regulation

### 4.1. Interaction between STAT3 and p53

In fibroblast cells, STAT3 binds to the promoter of p53, inhibiting its transcriptional expression and thus downregulating p53-reponsive genes [23]. There are multiple predicted STAT-binding sites within the human p53 promoter to which STAT3 can bind, but only one direct binding site exists for STAT3 to suppress p53 gene expression [23]. Alternatively, mutations at STAT3 binding sites partially restore p53 promoter activity [23]. In osteosarcoma cells, STAT3 and p53 protein interactions seem to be indirect because STAT3 protein binds to the p53-RELA complex, allowing it to interact with the miR-21 promoter [103]. Moreover, in prostate cancer cells, inactivation of both ELL-associated factor 2 (EAF2) and p53 can enhance STAT3 phosphorylation and drive tumorigenesis, and this regulation of STAT3 phosphorylation by EAF2 and/or p53 may involve multiple mechanisms [104]. Thus, it is not clear whether STAT3 protein solely exhibits direct interaction with p53 protein. Overall, these findings suggest that STAT3 exerts its effect mostly on the transcription of p53 and consequently on the protein level and cellular function of p53. 

### 4.2. STAT3 Inhibits p53-Mediated Apoptosis and Growth Arrest

The STAT3 oncogene is required as a downstream effector of SRC and promotes invasive phenotypes by suppressing p53 and p53-inducible protein caldesmon, an antagonist of podosome, which was found especially in invasive SRC-transformed cells in the context of metastatic cancer cells [105,106]. This inhibition was reversed by the expression of a STAT3 dominant negative [106]. Consistent with this finding, SRC-induced p53 downregulation mediated by STAT3 was abrogated when cells were introduced as a dominant-negative mutant of STAT3, resulting in the restoration of p53 expression [23]. Furthermore, cotransfection of v-SRC with the STAT3 expression vector would inhibit p53 expression. Another STAT3 activator, PDGF, could also reduce p53 expression [23]. Blocking STAT3 in human cells triggered p53-dependent apoptosis, increased p53 and p21 expression, and facilitated UV-induced growth arrest [23]. These results indicate that STAT3 activation could negatively regulate the p53 signaling pathway and its related effects on apoptosis and growth inhibition. 

### 4.3. p53 Regulates STAT3 Signaling in Cancer Cells

wtp53 may directly or indirectly inhibit STAT3 phosphorylation and subsequently inhibit STAT3 DNA binding activity. One possible mechanism is that wtp53 induces tyrosine phosphatase dephosphorylation of STAT3, such as PTEN and SHP2. The enhancement of p53 was found to attenuate STAT3 function and SRC-induced podosome formation by upregulating the tumor suppressor PTEN [106]. The functional loss of p53 results in intracellular reactive oxygen species (ROS) accumulation, leading to oxidation of the catalytic cysteine residues, and inactivation of the tyrosine phosphatase SHP2, which fails to dephosphorylate JAK2 and STAT3, thereby mediating persistent STAT3 activation [29]. Alternatively, wtp53 may inhibit upstream activators of STAT3, such as SRC, JAK2, or EGFR, to phosphorylate and activate STAT3 [21]. A recent report indicated a crosstalk between STAT3 and p53 to inversely regulate autophagy through the collaborative regulation of MAPK and PI3K/AKT signaling to control ovarian tumorigenesis and chemoresistance [107]. 

STAT3 is one of the downstream effectors of wtp53. This is the case in prostate and breast cancer cell lines where overexpression of wtp53 leads to a significant reduction in the phosphorylation at Tyr705 residue and DNA binding activity of STAT3 [21]. Another report indicated that p53 regulates long intergenic nonprotein coding transcripts via STAT3 signaling to promote cutaneous squamous carcinoma progression [108]. In addition, STAT3 is a part of the feedback loop miR-34a/CSF1R/STAT3, in which the miR-34a gene is a direct target of p53 in colorectal cancer [109]. 

**Figure 1:**
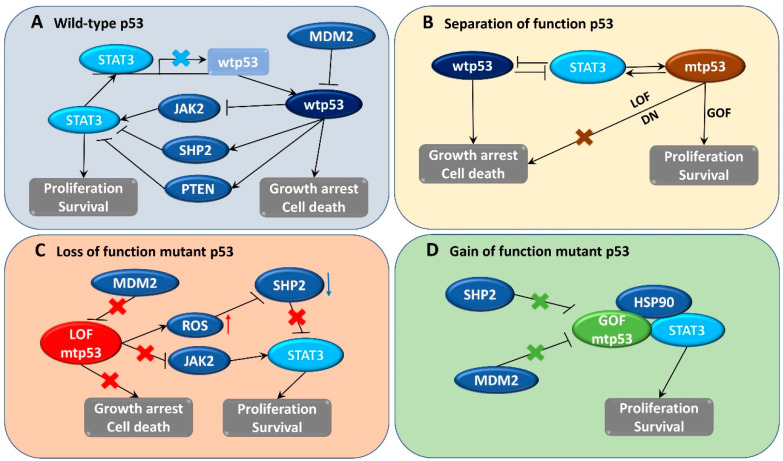
Schematic summarizing networks between signal transduction and activator of transcription 3 (STAT3) and different states of p53 in cancer cells. STAT3 usually functions as an oncogene to support cell proliferation and survival, whereas wild-type p53 (wtp53) exhibits tumor suppressive function by inducing cell growth arrest followed by cell death. (**A**) In cells expressing only wtp53, STAT3 binds to the promoter of wtp53 and inhibits its transcriptional expression, leading to reduction in the protein wtp53 and its related function. Wtp53 supports the expression of STAT3 inhibitors such as Src homology region 2 (SHP2) and phosphatase and tensin homolog (PTEN) or reduces the STAT3 upstream mediator janus kinase 2 (JAK2). (**B**) In early stages of tumorigenesis, the function of p53 is separated due to the appearance of p53 mutations. Mutant p53 (mtp53) may lose its original function (LOF) or exert dominant negative (DN) function against the wtp53 or gain function (GOF) to enhance tumor activity. STAT3 can control cell proliferation without the expression of functional wtp53. Alternatively, mtp53 promotes STAT3-mediated tumor cell growth or survival. (**C**) Functional loss of p53 results in reactive oxygen species (ROS) accumulation which disrupts SHP2 activity, thereby promoting persistent STAT3 expression. (**D**) GOF mtp53 interacts with a chaperone like heat shock protein (HSP90) to form a stable complex that prevents it from degradation and further promotes STAT3 activity. “MDM2” stands for mouse double minute 2, a wtp53 negative regulator.

In addition to wtp53, mtp53 also plays an important role in the activation of STAT3 signaling; mtp53 was also found to regulate cancer-associated fibroblast-specific factors such as α-SMA, FGF10, and CXCL12 through the STAT3 pathway [110]. LOF p53 contributes to JAK2-STAT3 signaling and promotes pancreatic tumor growth and stroma modification [29]. Phosphorylation of STAT3 correlated with LOF p53 mutation and patient survival time in human pancreatic tumors, and inhibition of this pathway could slow down tumor proliferation and formation of stroma [29]. Furthermore, ablation of GOF p53 inhibits STAT3-mediated tumor growth and invasion in colorectal cancer cells [28]. The physical interaction between stabilized abundant mtp53 and phosphorylated STAT3 prevents STAT3 from associating with its tyrosine phosphatase SHP2 and protects STAT3 from dephosphorylation and remains active [28]. In brief, wtp53 has an opposite effect to STAT3, whereas mtp53 seems to collaborate with STAT3 in the regulation of signaling related to cancer development (Figure 1).

### 4.4. Constitutive Activation of STAT3 Occurs in Cancer Cells Containing p53 Mutations 

As mentioned above, STAT3 is constitutively phosphorylated in several types of tumors, whereas the expression of p53 is not always stable and depends on the mutation status and specific cancer types. The status of STAT3 activation and p53 mutation in different cancer cell lines are summarized in Table 1. According to the feedback regulation between STAT3 and p53, it can be observed that cancer cells that persistently express active STAT3 also frequently express mtp53. However, the opposite inference seems to not be true because, in some cases, mtp53-harboring cells do not express constitutively active STAT3 like MDA-MB-453 breast cancer cells. In different colon cancer cell lines, the expression of p53 mutations at the hot-spot codon R248 was more stable and higher than at other mutation codon positions. Notably, knockdown of mtp53 reduced the phosphorylation of STAT3, whereas deletion of weakly expressed mtp53 did not change the STAT3 phosphorylation level [28]. In another case, STAT3 was found to be active in some p53 null cells, such as HCT116. The active expression seems not to be constitutive, as STAT3 might be activated and accumulated gradually during long-term culture under high-density conditions [111]. Although STAT3 expression in p53 null cells is not always stable, it is still consistent with the active STAT3 activity found in LOF mtp53-bearing cells. Collectively, these data suggest that hyperactive STAT3 expression might correlate favorably with the high expression of mtp53. 

## 5. Pharmacological Strategies to Target Both p53 and STAT3 Activities in Cancer Cells

Although several compounds targeting either STAT3 or p53 are in clinical trials, their success is still limited due to drug resistance in cancer cells, and the related resistance mechanisms have not been fully understood. Due to the frequent appearance of mtp53 in cancers, drugs that reactivate wtp53 function or degrade mtp53 increasingly enter clinical trials for cancer therapy. In case of STAT3 inhibitors, drugs targeting IL-6R/JAK/STAT3 are advantageous in the clinic, as this signaling pathway is very important in many human malignancies. Following numerous research papers regarding STAT3 and mtp53 inhibitory strategies, here we attempted to summarize selected drug candidates that have reported effects on STAT3 and p53 signaling with evidence of clinical trials (Table 2). 

Notably, niclosamide is a repurposed STAT3 inhibitor originally approved by the FDA for the treatment of intestinal tapeworm infections [157]. Niclosamide was reported to inhibit STAT3 activation and remove cancer cells containing dysfunctional p53 [157,158]. Several ongoing clinical studies are evaluating the effect of niclosamide on cancer treatment [159]. STAT3 and mtp53 have recently been shown to elicit positive feedback regulation involving HSP90 and the mevalonate pathway [160]. The HSP chaperone system plays a role in mtp53 stabilization by protecting mtp53 from degradation by E3 ubiquitin ligases [28]. Additionally, mtp53 could bind to the sterol biosynthesis gene promoter, implicating the supporting role of the mevalonate pathway in cancer with highly expressed mtp53 [161]. Therefore, inhibitors of HSP90 (ganetespib, onalespib, luminespib) or statins (atorvastatin, lovastatin, and simvastatin), which suppress the mevalonate pathway, are promising drugs with dual inhibitory effects for anticancer therapeutics. 

Other than drugs with identified clinical trials for cancer therapy listed in Table 2, there are other notable compounds that have been reported to have potential dual effects on STAT3 and p53. One example is AG490, a JAK2/STAT3 inhibitor, which could support the p53–p21 axis to induce Kaposi’s sarcoma-associated herpesvirus (KSHV) lytic cycle activation in lymphoma cells or downregulate HSP90 as well as mtp53 expression in glioblastoma and pancreatic cancer cells [160,162]. Another example is nitazoxanide, an FDA-approved antiprotozoal agent which was proved to have dual inhibitory effects on IL-6/JAK2/STAT3 and p53-dependent signaling pathways in colorectal cancer (CRC) cells [163]. Nitazoxanide is currently used with a spectrum of antibacterial drugs in a recruiting clinical trial to treat neoplasms (NCT02366884). Moreover, nitazoxanide could inhibit autophagy and support cell cycle arrest in glioblastoma, implicating its potential as an anticancer agent [164]. Some factors involving the STAT3–p53 regulatory loop can be further exploited to produce related biologic drugs, such as leukemia inhibitory factor (LIF) and human leukocyte antigen (HLA)-F adjacent transcript 10 (FAT10). It was indicated that LIF is a p53-negative regulator that downregulated p53 expression level and function through activation of STAT3 [165]. In another study, FAT10 was determined as a mediator in the link between STAT3 and p53; overexpression of STAT3-regulated FAT10 could suppress p53 transcriptional activity [166]. Thus, inhibitors targeting these factors could become potential dual target agents in cancer treatment.

## 6. Conclusions

Several strategies utilize conventional small-molecule drugs to target either STAT3 or p53 and their related signaling pathways to prevent cancer development. Growing evidence still promotes STAT3 or p53 as potential molecular targets for cancer treatment, despite clinical outcomes that might be affected by drug resistance. Therefore, co-targeting STAT3 and p53 could be a promising approach to overcome drug resistance and speed up clinical trials. The STAT3–p53 regulatory loop consists of two aspects: negative regulation between STAT3 and wtp53 and positive regulation between STAT3 and mtp53. Not only direct STAT3 or p53 target molecules, but also factors (HSP90, MDM2) or pathways (such as the mevalonate pathway) involved in this feedback loop could be exploited to regulate both STAT3 and p53-mediated signaling for cancer therapies. More efforts in drug development are necessary, and more evidence on the efficacy of drug combination treatment should be provided to facilitate the translation of available inhibitors into clinical trials.

## Figures and Tables

**Table 1:** The status of p53 mutation and transcription 3 (STAT3) constitutive activation in human cancer cell lines.

Cancer Types	Cell Lines	p53 Status	STAT3 Status	Refs.
Colon	LS174T	Wild-type	No	[112,113]
	SW48	Wild-type	No	[114,115]
	SW480	Mutant (R273H, P309S) ^a^	Active	[28,116,117]
	LS123	Mutant (R175H) ^a^	Active	[118]
	LS1034	Mutant (G245S)	Active	[28]
	COLO320DM	Mutant (R248W)	Active	[28]
	WiDr	Mutant (R273H)	Low	[112,119]
	HCT116	Wild-type	Active	[115,120,121]
	HT-29	Mutant (R273H)	Active	[116,117,122]
	SW620	Mutant (R273H, P309S) ^a^	No	[121,123]
	SW1463	Mutant (R248Q) ^a^	Active	[28]
	SW837	Mutant (R248W) ^a^	Active	[28]
	DLD-1	Mutant (S241F)	No	[28,121]
Breast	MDA-MB-231	Mutant (R280K) ^a^	Active	[38,39,124]
	MDA-MB-361	Mutant (E56X)	No	[38,125]
	MCF-7	Wild type	Active	[38,39,126]
	MDA-MB-453	Mutant (T387S)	No	[38,126,127]
	MDA-MB-435S	Mutant (G266E)	Active	[38,125]
	MDA-MB-468	Mutant (R273H) ^a^	Active	[124,125,127]
	MDA-MB-436	Mutant (E204fsX45)	Active	[125,126]
	AU565	Mutant (R175H) ^a^	Active	[121,128]
	SK-BR-3	Mutant (R175H) ^a^	Active	[38,125,129]
	HCC70	Mutant (R248Q)	Active	[126,130,131]
	BT-549	Mutant (R249S) ^a^	Active	[125,126]
	HCC38	Mutant (R273L)	Active	[130,132,133]
	T47D	Mutant (L194F) ^a^	Active	[38,125,126]
Lung	BEAS-2B	Wild type ^b^	No	[134]
	A549	Wild type	Active	[121,135]
	NCI-H596	Mutant (G245C)	Active	[136]
	NCI-H1299	Null	No	[135]
	NCI-H1975	Mutant (R273H)	Active	[130,137]
	NCI-H1882	Wild type ^b^	Active	[138]
	NCI-H1417	Mutant (R175L) ^b^	Active	[138]
	NCI-H719	Mutant (R248Q) ^b^	Active	[138]
	NCI-H1105	Mutant (R249S) ^b^	Active	[138]
	NCI-H1048	Mutant (R273C) ^b^	Active	[130,138]
Pancreatic	SW1990	Wild type ^b^	Active	[139]
	SU.86.86	Mutant (G245S) ^b^	No	[140]
	BXPC-3	Mutant (Y220C) ^a^	No	[140]
	PANC-1	Mutant (R273H) ^a^	Active	[39,124]
	MIA-PaCa-2	Mutant (R248W/R273H) ^a^	Active	[140]
	Colo 357	Wild-type	Active	[124]
Prostate	DU-145	Mutant (P223L/V274F) ^a^	Active	[129,141]
	PC-3	Null	Active	[129,141]
	LNCaP	Wild-type	Active	[129,141]
Ovary	SKOV3	Mutant (H179R)	Active	[122,136,142,143]
	OVCAR3	Mutant (R248Q)	Active	[142,143]
	OVCA420	Mutant (R273H)	Active	[142,144]
	OVCA433	Mutant (E258K)	Active	[142,145]
	OVCA429	Wild-type	Active	[144]
	Caov-3	Mutant (Q136 ^c^)	Active	[146,147]
	A2780	Wild-type	No	[147,148]
	MDAH 2774	Mutant (R273H)	Active	[147,148]
	SW626	Mutant (G262V) ^b^	No	[147]
Melanoma	SK-MEL-2	Mutant (G245S)	Active	[122,149]
	SK-MEL-28	Mutant (L145R)	Active	[122,149]
	SK-MEL-5	Wild-type	Active	[122,149]
	Malme-3M	Wild-type	Active	[122,150]
	MeWo	Mutant (E258K, Q317 ^c^) ^b^	Active	[149]
Leukemia	HL-60	Null ^b^	Active	[151,152]
	K-562	Mutant (Q136fs*13) ^b^	Active	[151,152]
	Kasumi-1	Mutant (R248Q) ^b^	Active	[151]
	MOLT-4	Mutant (R306 ^c^) ^a^	Active	[152]
	RPMI-8226	Mutant (E285K) ^a^	No	[153,154]
	CCRF-CEM	Mutant (R248Q, R175H) ^a^	Active	[155]

^a^ p53 mutation status was identified from COSMIC database [156]. ^b^ p53 mutation status was identified from ATCC’s cell profile (https://www.atcc.org). ^c^ Mutation type: chain termination at that codon. fs: Frame shift.

**Table 2:** Search for potential compounds targeting STAT3 and p53.

Inhibitor	Target	Mechanism	Phase ^a^	Potential Effects on STAT3/p53	Refs.
Napabucasin (BBI-608)	STAT3	Inhibits gene transcription driven by Stat3	I/II (Advanced malignancies) III (CRC)	BBI-608 blocks mtp53 (R248Q)-mediated STAT3 activation	[28,167]
Celecoxib ^b^	STAT3	Binds to the three sub-pockets of STAT3 SH2; Inhibits IL-6/STAT3 signaling pathway	III (Breast cancer) II (Lung cancer, Glioblastoma)	Treatment with bortezomib and celecoxib induced apoptosis in p53-degraded cancer cells. Inhibition of COX-2 in colon cancer cell lines by celecoxib increases the nuclear localization of active p53. Celecoxib enhances irradiation-induced apoptosis by p53 signaling. Inhibition of STAT3 pathways by celecoxib induced autophagy, which promoted the degradation of mtp53.	[168,169,170,171,172,173]
Ruxolitinib ^b^ (INC424)	STAT3	Targets JAK2/STAT3 axis	IV (Myelofibrosis) II (Leukemia, Lymphoma)	Ruxolitinib blocks mtp53 (R248Q)-mediated STAT3 activation	[28,71]
Cetuximab ^b^	STAT3	Targets EGFR/STAT3 axis	II (Metastatic CRC) I (Squamous Cell Carcinoma, Head and Neck)	The loss of p53 was associated with acquired resistance to EGFR inhibitor. Down-regulation of p53 and up-regulation of EGFR expression increase the sensitivity to cetuximab. Cetuximab administered after oxaliplatin reduces STAT3 phosphorylation and up-regulates p53 protein level.	[174,175,176,177]
Sorafenib ^b^	STAT3	Targets JAK2/STAT3 axis	IV (Advanced hepatocellular carcinoma) III (Renal cell carcinoma; Non small cell lung carcinoma) II (CRC metastatic; Thyroid cancer)	Sorafenib kills cancer cells by activating PUMA, a p53 target and a BH3-only Bcl-2 family protein. Sorafenib has synergistic effects with genistein to increase apoptosis through up-regulation of p53 and p21.	[69,178,179]
Sunitinib ^b^	STAT3	Acts as VEGF, PDGFR inhibitor; inhibits tyrosine kinase phosphorylation of STAT3	II (Renal carcinoma, pancreatic neuroendocrine tumor metastatic)	Sunitinib treatment increased p53 levels in renal cell carcinoma xenografts Sunitinib induces cellular senescence through p53/DEC1 signaling activation in renal cell carcinoma cells	[70,180,181]
KX2-391	STAT3	Inhibits SRC-STAT3 axis	II (Bone-metastatic, castration-resistant prostate cancer) I (Lymphoma)	KX2-391 has antiproliferative effect by inducing p53 expression in SRC3T3 and HT29 cells	[182,183]
Niclosamide ^b^	STAT3	Inhibits STAT3 activation, nuclear translocation and transactivation	I (Prostate carcinoma)	Niclosamide can activate p53 function in wild-type cells while reducing the growth of p53-deficient cells and p53 mutant patient-derived ovarian xenografts	[157,158]
PRIMA-1^Met^ (APR-246)	p53	Converts to methylene quinuclidinone which binds to thiol group of mtp53 and restores wtp53	II (High-grade serous ovarian cancer) I (Hematologic, prostatic neoplasms)	APR-246 exhibited synergistic effect with piperlongumine to induce cell death in mtp53 HNSCC. Piperlongumine has been identified as a potential direct STAT3 inhibitor against breast cancer.	[184,185,186]
Ganetespib	p53	Inhibits HSP90 and degrades mtp53	II (Lung, colon, rectal cancer, gastrointestinal stromal tumor, breast cancer, melanoma)	Phosphorylation level of STAT3 correlated with HSP90 inhibitor resistance in TNBC cells. Ganatespib inhibited pancreatic cancer cell growth via down-regulation of JAK2-STAT3 pathway.	[187,188,189]
Onalespib (AT13387)	p53	Inhibits HSP90 and degrades mtp53	I (Advanced malignant solid neoplasm)	AT13387 reduced EGFR/STAT3 in nasopharyngeal carcinoma cells. AT13387 inhibited IL-6-mediated STAT3 phosphorylation in myeloma and breast carcinoma cells.	[190,191,192]
Luminespib (AUY922)	p53	Inhibits HSP90 and degrades mtp53	II (Non-small cell lung cancer)	NVP-AUY922 enhances TRAIL-induced apoptosis by down-regulating JAK2-STAT3-Mcl-1 signal transduction pathway in colorectal cancer cells. Treatment with NVP-AUY922 negatively affected IL-6-mediated STAT3 phosphorylation. AUY922 treatment disrupts the association between HSP90 and its client proteins (JAK2, STAT3) and reduced the levels of STAT3. AUY922 treatment inhibited STAT3 activity in chronic lymphocytic leukemia cells.	[193,194,195,196,197]
Atorvastatin ^b^	p53	Inhibits HMG-CoA reductase, disrupts mtp53-HSP90 complex	II (Prostatic neoplasms)	Atorvastatin treatment reduced phosphorylation of STAT3 in head and neck squamous cell carcinoma. The combined use of atorvastatin and aspirin attenuated STAT3 phosphorylation in the treatment of prostate cancer. Atorvastatin induced senescence of hepatocellular carcinoma through downregulation of IL-6/STAT3 pathway.	[198,199,200,201]
Lovastatin ^b^	p53	Inhibits HMG-CoA reductase, disrupts mtp53-HSP90 complex	II (Prostate cancer)	Lovastatin inhibited mevalonate pathway that reduced mtp53 expression, and inhibited STAT3 phosphorylation in glioblastoma and pancreatic cancer cells	[160]
Simvastatin ^b^	p53	Inhibits HMG-CoA reductase, disrupts mtp53-HSP90 complex, degrades mtp53	III (Gastric cancer) II (Breast cancer)	Simvastatin reduced renal cancer cell growth and metastasis through inhibition of JAK2/STAT3 pathway. Simvastatin induced growth arrest by suppressing STAT3/SKP2 in HCC cells.	[161,202,203]

^a^ Completed clinical trials (https://clinicaltrials.gov/). ^b^ FDA-approved drugs (https://www.fda.gov/).

## Abbreviations

APAF1	Apoptotic peptidase activating factor 1
BAX	BCL2 associated X
DDB2	Damage specific DNA binding protein 2
E2F7	E2F transcription factor 7
ERCC5	ERCC excision repair 5
FANCC	FA complementation group C
GADD45	Growth arrest and DNA damage inducible
KSHV	Kaposi’s sarcoma-associated herpesvirus
MGMT	O-6-methylguanine-DNA methyltransferase
MSH2	MutS homolog 2
PML	Promyelocytic leukemia
POLK/POLH	DNA polymerase kappa/ DNA polymerase eta
PUMA	p53 upregulated modulator of apoptosis
SLC7A11	Solute carrier family 7 member 11
TIGAR	TP53 induced glycolysis regulatory phosphatase
XPC	Xeroderma pigmentosum, complementation group C
